# Serum Wisteria Floribunda Agglutinin-Positive Mac-2 Binding Protein Values Predict the Development of Hepatocellular Carcinoma among Patients with Chronic Hepatitis C after Sustained Virological Response

**DOI:** 10.1371/journal.pone.0129053

**Published:** 2015-06-12

**Authors:** Ryu Sasaki, Kazumi Yamasaki, Seigo Abiru, Atsumasa Komori, Shinya Nagaoka, Akira Saeki, Satoru Hashimoto, Shigemune Bekki, Yuki Kugiyama, Atsushi Kuno, Masaaki Korenaga, Akira Togayachi, Makoto Ocho, Masashi Mizokami, Hisashi Narimatsu, Tatsuki Ichikawa, Kazuhiko Nakao, Hiroshi Yatsuhashi

**Affiliations:** 1 Clinical Research Center, National Hospital Organization, Nagasaki Medical Center, Omura, Japan; 2 Research Center for Medical Glycoscience, National Institute of Advanced Industrial Science and Technology, Tsukuba, Japan; 3 The Research Center for Hepatitis and Immunology, National Center for Global Health and Medicine, Ichikawa, Japan; 4 Department of Gastroenterology and Hepatology, Graduate School of Biomedical Sciences, Nagasaki University, Nagasaki, Japan; Kaohsiung Medical University Hospital, Kaohsiung Medical University, TAIWAN

## Abstract

Measurement of *Wisteria floribunda *agglutinin-positive human Mac-2 binding protein (WFA^+^-M2BP) in serum was recently shown to be a noninvasive method to assess liver fibrosis. The aim of this study was to evaluate the utility of serum WFA^+^-M2BP values to predict the development of hepatocellular carcinoma (HCC) in patients who achieved a sustained virological response (SVR) by interferon treatment. For this purpose, we retrospectively analyzed 238 patients with SVR who were treated with interferon in our department. Serum WFA^+^-M2BP values were measured at pre-treatment (pre-Tx), post-treatment (24 weeks after completion of interferon; post-Tx), the time of HCC diagnosis, and the last clinical visit. Of 238 patients with SVR, HCC developed in 16 (6.8%) patients. The average follow-up period was 9.1 years. The cumulative incidence of HCC was 3.4% at 5 years and 7.5% at 10 years. The median pre-Tx and post-Tx WFA^+^-M2BP values were 1.69 (range: 0.28 to 12.04 cutoff index (COI)) and 0.80 (range: 0.17 to 5.29 COI), respectively. The WFA^+^-M2BP values decreased significantly after SVR (*P* < 0.001). The median post-Tx WFA^+^-M2BP value in patients who developed HCC was significantly higher than that in patients who did not (*P* < 0.01). Multivariate analysis disclosed that age (> 60 years), sex (male), pre-Tx platelet count (< 15.0×10^3^/μL), and post-Tx WFA^+^-M2BP (> 2.0 COI) were associated with the development of HCC after SVR.

**Conclusion:**

Post-Tx WFA^+^-M2BP (> 2.0 COI) is associated with the risk for development of HCC among patients with SVR. The WFA^+^-M2BP values could be a new predictor for HCC after SVR.

## Introduction

Hepatocellular carcinoma (HCC) is one of the most common malignant tumors in the world [1]. Chronic hepatitis C virus (HCV) infection is a major cause of HCC. Millions of people are persistently infected with HCV globally [2–4] and these individuals are at high risk of developing HCC [5–7]. Several studies have demonstrated that interferon (IFN) treatment in chronic hepatitis C patients reduces the risk for progression of liver disease, HCC, liver-related death, and all-cause mortality [8–13], especially in patients who exhibit a sustained virological response (SVR). However, some risk for HCC—albeit a small one—remains even after achieving viral eradication [10,14–19]. Several factors have been reported to affect HCC development among patients with SVR.

Recently, an assay for the measurement of *Wisteria floribunda* agglutinin-positive human Mac-2 binding protein (WFA^+^-M2BP) was reported as a novel, noninvasive, and rapid bedside method to assess liver fibrosis [20]. M2BP has been shown to have multibranching and sialylated N-glycans. WFA is considered to recognize the GalNAc residue of N-glycans and O-glycans or the clustered LacNAc (Gal-GlcNAc) structure. Currently, we are analyzing the glycan structures of WFA^+^-M2BP in detail using MS-based technology [21]. Glycans can reflect the differentiation stage of cells but not necessarily the level of cellular damage, and therefore they can be very effective markers for chronic disease. Several reports performed with proteome analysis have identified Mac-2 binding protein as a potential marker of liver fibrosis progression [22–25]. Kuno et al. were the first to report that a rapid and simple glycan-based immunoassay for WFA^+^-M2BP can quantify fibrosis [20,26]. On the other hand, we reported that AFP and WFA^+^-M2BP values are noninvasive predictive markers for the development of HCC in patients with HCV [27,28]. In this report we evaluated the utility of WFA^+^-M2BP values to predict the development of HCC in patients who had achieved SVR after IFN treatment.

## Patients and Methods

### Patients

From December 1989 to December 2010, a total of 601 consecutive HCV patients who received IFN treatment and achieved SVR at the National Hospital Organization Nagasaki Medical Center were enrolled in this retrospective study. The diagnosis of chronic HCV infection was based on continuous positivity for both anti-HCV by a second or third-generation enzyme-linked immunoadsorbent assay (ELISA) and positivity for serum HCV RNA by polymerase chain reaction (PCR). Before treatment, HCC was definitively ruled out either by ultrasonography (US), dynamic computed tomography (CT), or magnetic resonance imaging (MRI) on enrollment. Exclusion criteria for this study were: (1) positivity for hepatitis B surface antigen; (2) positivity for human immunodeficiency virus; (3) autoimmune hepatitis or primary biliary cirrhosis; (4) a shorter follow-up period (< 12 months) after the completion of IFN treatment; (5) a history of HCC at the time of IFN treatment; (6) development of HCC within 12 months after the completion of IFN treatment; (7) administration of low dose long-term IFN treatment; and (8) absence of properly stored serum samples or insufficient archival material. After the exclusions, 238 patients who achieved SVR were analyzed retrospectively for the risk factors of HCC.

For all patients in our cohort, a blood sample was taken on the days of the administration of IFN treatment (pre-treatment; pre-Tx), 24 weeks after completion of IFN treatment (post-treatment; post-Tx), and on the days of HCC diagnosis and last clinical visit. All separated serum samples were stored at -20°C until use. Medical histories, along with the results of routine tests for blood cell counts, liver biochemistry and HCV viral load/genotype at the time of IFN treatment and thereafter, were retrieved from medical records. Complete blood cell counts and biochemical tests were performed using automated procedures in the clinical pathologic laboratories of our hospital.

### Histological evaluation

Liver biopsies were undertaken using fine-needle aspiration (16G or 18G sonopsy) guided by US. Liver tissue specimens were fixed in 10% formalin, embedded in paraffin, and stained with hematoxylin and eosin. The histological assessment was made by two independent pathologists according to the classification of Desmet et al. [29].

### Interferon treatment

Among the 238 patients, 123 received IFN monotherapy for 24 weeks, 28 patients received pegylated (PEG-)IFN monotherapy for 48 weeks, and 87 patients received IFN plus ribavirin or PEG-IFN plus ribavirin combination therapy for 48–72 weeks.

### HCV RNA and HCV genotypes

The presence of HCV RNA was determined by reverse transcriptase (RT-) PCR using a commercial kit (Amplicor HCV; Roche Diagnostic Systems, Basel, Switzerland). Genotypes of HCV were determined by RT-PCR with genotype-specific primers (HCV RNA core genotype; Roche Diagnostics, Tokyo, Japan) [30,31]. In patients treated before the availability of PCR, the presence of HCV RNA was investigated by using sera stored at -20°C.

### Definitions of response to interferon treatment

SVR was defined as the absence of detectable HCV RNA at 24 weeks after the end of IFN treatment. There was no relapse of viremia after 24 weeks among the patients who achieved SVR.

### Measurement of Wisteria floribunda agglutinin-positive human Mac-2 binding protein (WFA^+^-M2BP)

WFA^+^-M2BP quantification was performed based on a lectin-antibody sandwich immunoassay using a fully automatic HISCL-2000i immunoanalyzer (Sysmex Co., Hyogo, Japan) [20]. The measured values of WFA^+^-M2BP conjugated to WFA were indexed with the values obtained using the following equation:

$$\begin{array}{l} \text{Cutoff index }\left(\text{COI}\right)\text{ = }\left({\left[{\text{WFA}}^{\text{+}}\text{-M2BP}\right]}_{\text{sample}}-\;{\left[{\text{WFA}}^{\text{+}}\text{-M2BP}\right]}_{\text{NC}}\right)\text{ / }\left({\left[{\text{WFA}}^{\text{+}}\text{-M2BP}\right]}_{\text{PC}}\right)\text{ - }\\ {\left[{\text{WFA}}^{\text{+}}-\text{-M2BP}\right]}_{\text{NC}} \end{array}$$

Here, [WFA^+^-M2BP]_sample_ represents the WFA^+^-M2BP count of the serum sample (PC, positive control; NC, negative control). The positive control was supplied as a calibration solution preliminarily standardized to yield a COI value of 1.0 [26].

### Follow-up and diagnosis of hepatocellular carcinoma

All patients were followed up at an interval of 1–12 months by measurement of blood count and liver biochemistry, along with quantitative detection of HCV RNA, AFP, AFP-L3, and DCP. Diagnostic imaging either by US, CT, or MRI was performed at least once per year. A diagnosis of HCC was made based on positive results of typical vascular patterns, as revealed by either contrast-enhanced CT, contrast-enhanced MRI or angiography. Otherwise, the pathological diagnosis was made by fine-needle biopsy of space-occupying lesions detected in the liver.

### Ethical considerations

Informed consent to utilize medical records and specimens was obtained from each patient. We obtained the written consent of participants at the time of serum collection. These processes and the study protocol were approved by the Ethical Committee of National Hospital Organization Nagasaki Medical Center (confirmation number: 25102), and conformed with the 1975 Declaration of Helsinki and the Japanese Ethical Guidelines for Clinical Research (Ministry of Health, Labor, and Welfare of Japan, Ethical Guidelines for Clinical Research, 2008). Our research is available on the National Hospital Organization Nagasaki Medical Center website (http://www.nagasaki-mc.jp/).

### Statistical analysis

Continuous variables (AST, ALT, albumin, total bilirubin, γ-GTP, fasting blood sugar, HbA1c, triglyceride, total cholesterol, BMI, platelet counts, AFP, WFA^+^-M2BP) were dichotomized with respect to the median value or clinically meaningful values in the multivariate analysis. Statistical analysis was performed using a Wilcoxon signed rank test and Mann-Whitney U-test. To estimate the cumulative risk of developing HCC, the Kaplan-Meier method and the log-rank test were used. Cox proportional hazards regression analysis was performed to evaluate risk factors for HCC. The diagnostic performances of WFA^+^-M2BP and AFP for censored development of HCC were assessed by examining the area under the time-dependent receiver operating characteristic (ROC) curves (AUROC) [32]. Inclusion of variables was assessed using a stepwise selection method. A *P* value of 0.05 was considered statistically significant. Data analysis was performed with SPSS ver. 22.0 (SPSS, Chicago, IL).

## Results

### Patient characteristics

The baseline characteristics of the 238 patients are summarized in Table 1. The median age was 55.0 years; 147 (61.8%) patients were male; and 104 (43.7%), 68 (28.6%), 42 (17.6%) and 24 (10.1%) patients were diagnosed histologically with fibrosis stage F1, F2, F3 and F4, respectively. The median value of the pre-Tx platelet count was 16.0 (range: 6.4 to 33.2 ×10^3^/μL) and that of the post-Tx platelet count was 16.8 (range: 6.5 to 36.3 ×10^3^/μL). The median value of pre-Tx AFP was 5 (range: 1 to 200 ng/mL) and that of post-Tx AFP was 3 (range: 1 to 46 ng/mL). The average follow-up period was 9.1 years.

**Table 1:** Characteristics of Patients Enrolled in the Present Study.

Factors	Value
Patients, n	238
Age, year	55.0 (18–75)
Male, n (%)	147 (61.8)
BMI (kg/m^2^)	23.20 (16.7–34.9)
Alcohol intake (> 20g/day), n (%)	64 (26.9)
Fibrosis stage, n (%) F 1/2/3/4	104 (43.7)/68 (28.6)/42 (17.6)/24 (10.1)
Steatosis (≥ 10%), n (%)	25(10.5)
Pre-Tx platelet counts (×10^3^/μL)	16.0 (6.4–33.2)
Post-Tx platelet counts (×10^3^/μL)	16.8 (6.5–36.3)
Albumin (g/dL)	4.30 (2.9–5.5)
Pre-Tx AST (IU/mL)	60.0 (12–365)
Post-Tx AST (IU/mL)	20.0 (10–54)
Pre-Tx ALT (IU/mL)	100.0 (12–519)
Post-Tx ALT (IU/mL)	17.0 (7–64)
γ-GTP (IU/L)	37.0 (7–1790)
T. Bilirubin (mg/dL)	0.70 (0.3–1.9)
HbA1c (%)	5.70 (4.4–8.1)
Pre-Tx AFP (ng/mL)	5.0 (1–200)
Post-Tx AFP (ng/mL)	3.0 (1–46)
Pre-Tx WFA^+^-M2BP (COI)	1.70 (0.28–12.04)
Post-Tx WFA^+^-M2BP (COI)	0.80 (0.17–5.29)
HCV serogroup, n (%)	
1	111 (46.6)
2	101 (42.4)
Unknown	26 (11.0)
IFN regimen, n (%)	
IFN monotherapy	123 (51.6)
PEG-IFN monotherapy	28 (11.8)
IFN/PEG-IFN+RBV	87 (36.6)
Observation period, years	9.1 (5.6) *

Data are given as the medians with ranges.

*Results are expressed as the means ± standard deviation. Unless otherwise indicated, data were collected at pre-treatment (before administration of IFN therapy; pre-Tx). Several biochemical measurements were made at both pre-Tx and post-treatment (24 weeks after completion of IFN therapy; post-Tx).

Abbreviations: AST, aspartate aminotransferase; ALT, alanine aminotransferase; γ-GTP, γ-glutamyl transpeptidase; HbA1c, glycated hemoglobin; BMI, body mass index; AFP, α-fetoprotein; HCV, hepatitis C virus; PEG-IFN, pegylated interferon; RBV, ribavirin.

### Cumulative incidence of HCC

During the follow-up period, HCC developed in 16 (6.8%) of the 238 patients. The cumulative incidences of HCC at 5 and 10 years were 3.4% and 7.5%, respectively.

### Risk factors for HCC

Univariate analysis demonstrated factors that increase the risk for HCC development after SVR. Cox regression analysis was performed on 20 variables: age, sex, BMI, alcohol intake, fibrosis stage, degree of steatosis, pre-Tx platelet counts, post-Tx platelet counts, albumin, pre-Tx AST, post-Tx AST, pre-Tx ALT, post-Tx ALT, γ-GTP, T.bilirubin, HbA1c, pre-Tx AFP, post-Tx AFP, pre-Tx WFA^+^-M2BP, post-Tx WFA^+^-M2BP. Cutoff values for AFP and WFA^+^-M2BP were determined by time-dependent ROC analysis as 5 ng/ml and 2.0 COI, respectively.

The following seven factors were identified as posing an increased risk for HCC by the univariate analysis: age, fibrosis stage, albumin, pre-Tx platelet count, post-Tx platelet count, post-Tx AFP, and post-Tx WFA^+^-M2BP (Table 2).

**Table 2:** Factors Associated with Hepatocellular Carcinoma.

		Hazard Ratio (95% CI)	*P*	Hazard Ratio (95% CI)	*P*
Univariate analysis				Multivariate analysis	
Age (year)	≤ 60	1		1	
	> 60	6.09 (2.03–18.26)	0.001	5.42 (1.59–18.47)	0.007
Sex	Female	1		1	
	Male	1.12	0.290	4.71 (1.23–17.92)	0.023
BMI (kg/m^2^)	≤ 23.0	1			
	> 23.0	2.09 (0.71–6.11)	0.167		
Alcohol intake (g/day)	≤ 20	1			
	> 20	1.60 (0.58–4.42)	0.364		
Fibrosis stage	F1/2	1			
	F3/4	4.62 (1.67–12.81)	0.003		
Steatosis (%)	≤ 10	1			
	> 10	1.12 (0.30–5.69)	0.561		
Pre-Tx Platelet counts	≥ 15.0	1		1	
(×10^3^/μL)	< 15.0	4.75 (1.52–14.79)	0.007	4.72 (1.45–15.30)	0.010
Post-Tx Platelet counts	≥ 15.0	1			
(×10^3^/μL)	< 15.0	3.21 (1.20–12.96)	0.011		
Albumin (g/dL)	≥ 4.0	1			
	< 4.0	3.40 (1.22–9.45)	0.018		
Pre-Tx AST (IU/mL)	≤ 60	1			
	> 60	2.13 (0.74–6.14)	0.146		
Post-Tx AST (IU/mL)	≤ 20	1			
	> 20	2.33 (0.30–18.29)	0.473		
Pre-Tx ALT (IU/mL)	≤ 80	1			
	> 80	2.16 (0.79–5.83)	0.128		
Post-Tx ALT (IU/mL)	≤ 15	1			
	> 15	2.44 (0.72–8.31)	0.180		
γ-GTP (IU/L)	≤ 40	1			
	> 40	1.73 (0.60–4.99)	0.297		
T. Bilirubin (mg/dL)	≤ 1.0	1			
	> 1.0	1.66 (0.37–7.35)	0.481		
HbA1c (%)	≤ 5.5	1			
	> 5.5	1.07 (0.26–4.46)	0.929		
Pre-Tx AFP (ng/mL)	≤ 5.0	1			
	> 5.0	2.50 (0.89–7.06)	0.079		
Post-Tx AFP (ng/mL)	≤ 5	1			
	> 5	4.60 (1.53–13.84)	0.006		
Pre-Tx WFA^+^-M2BP	≤ 2.0	1			
(COI)	> 2.0	1.37 (0.49–3.77)	0.551		
Post-Tx WFA^+^-M2BP	≤ 2.0	1		1	
(COI)	> 2.0	7.30 (2.20–24.17)	0.001	5.71 (1.66–19.57)	0.006

Hazard ratios for the development of hepatocellular carcinoma were calculated by Cox proportional hazards analysis.

Abbreviations: AST, aspartate aminotransferase; ALT, alanine aminotransferase; γ-GTP, γ-glutamyl transpeptidase; HbA1c, glycated hemoglobin; BMI, body mass index; AFP, α-fetoprotein; HCV, hepatitis C virus; WFA^+^-M2BP, *Wisteria floribunda* agglutinin-positive human Mac-2 binding protein.

Multivariate analysis was performed on these seven factors, and the following four factors were identified as independent risk factors: age (> 60 years, HR 5.42, 95% CI = 1.59–18.47, *P* = 0.007), sex (male, HR 4.71, 95% CI = 1.23–17.92, *P* = 0.023), pre-Tx platelet count (< 15.0×10^3^/μL, HR 4.72, 95% CI = 1.45–15.30, *P* = 0.010), and post-Tx WFA^+^-M2BP values (> 2.0 COI, HR 5.71, 95% CI = 1.66–19.57, *P* = 0.006).

### Development of HCC

**Fig 1:**
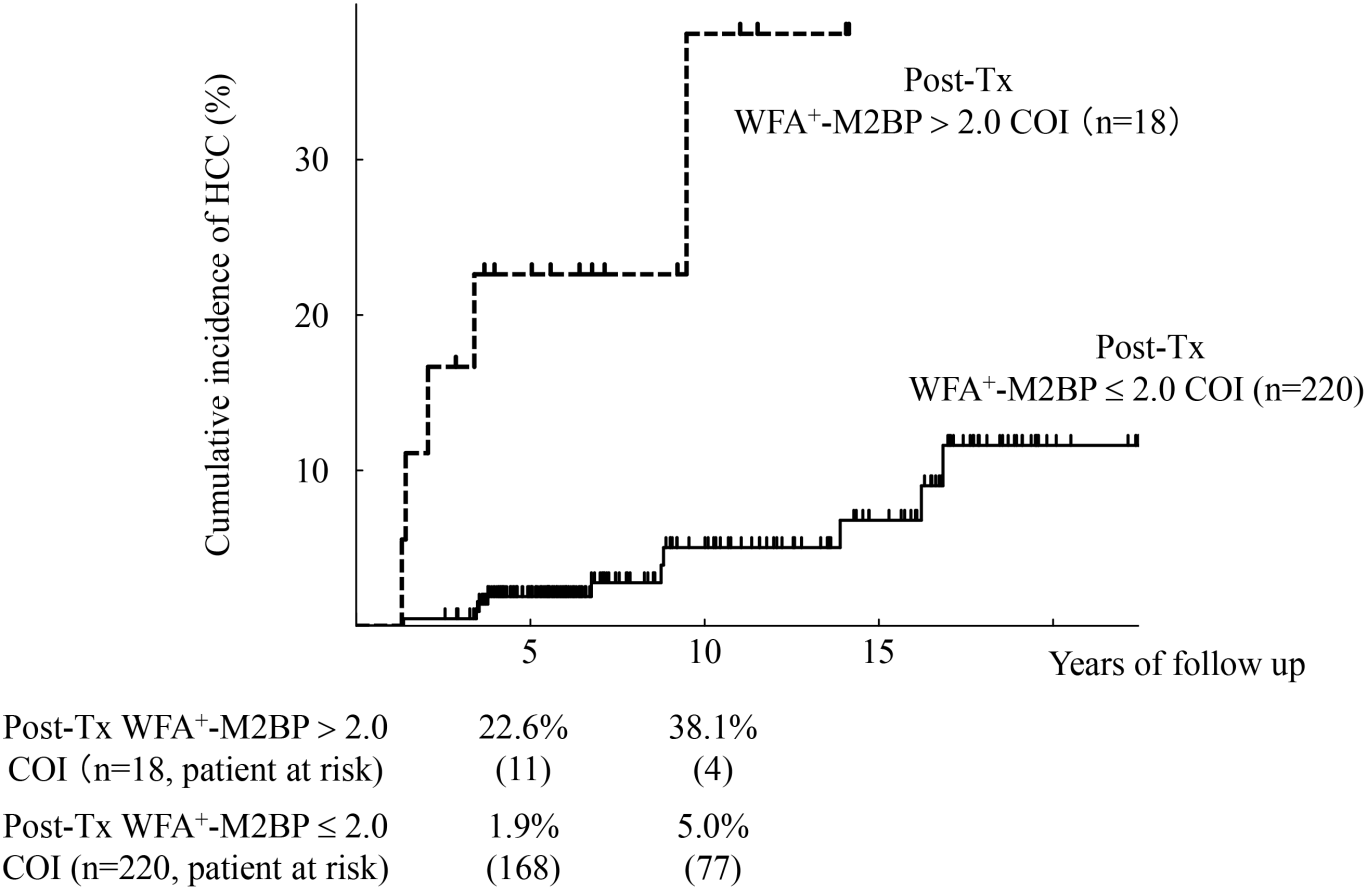
Cumulative incidence of hepatocellular carcinoma (HCC) according to post-treatment WFA^+^-M2BP values. Cumulative incidences of HCC according to post-treatment WFA^+^-M2BP values were analyzed using the Kaplan-Meier method. The black solid and dotted lines indicate the stratified post-treatment WFA^+^-M2BP values with a COI ≤ 2.0 and a COI > 2.0, respectively. The incidence rate differed significantly between the two groups (*P* < 0.0001 by the log-rank test). The numbers of patients at risk at each time point are shown below the graphs.

To evaluate the relation between post-Tx WFA^+^-M2BP values and development of HCC, we characterized 238 patients who achieved SVR with respect to their post-Tx WFA^+^-M2BP values. Fig 1 shows the cumulative risk of HCC and the post-Tx WFA^+^-M2BP values. The 5 and 10-year cumulative risks of HCC were 1.9% and 5.0% in the 18 patients with post-Tx WFA^+^-M2BP > 2.0 COI (post-Tx WFA^+^-M2BP > 2.0 COI group), and 22.6% and 38.1% in the 220 patients with post-Tx WFA^+^-M2BP ≤ 2.0 COI (post-Tx WFA^+^-M2BP ≤ 2.0 COI group). The incidence rates were significantly higher in the post-Tx WFA^+^-M2BP > 2.0 COI group (*P* < 0.0001 by the log-rank test).

**Fig 2:**
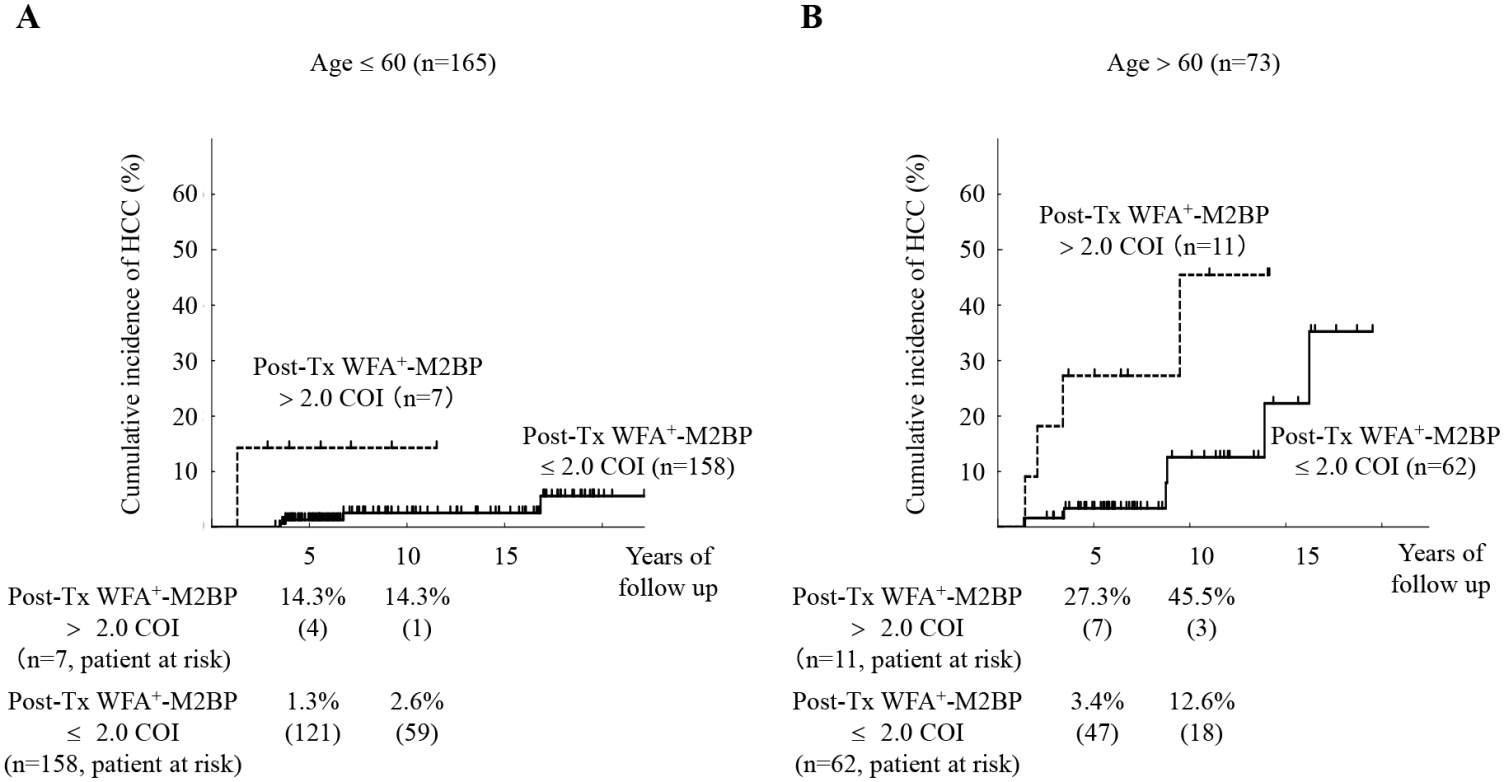
Cumulative incidence of hepatocellular carcinoma (HCC) according to post-treatment WFA^+^-M2BP values, stratified by age. (A): Age ≤ 60 years (n = 165). (B): Age > 60 years (n = 73). Cumulative incidences of HCC according to post-treatment WFA^+^-M2BP values were analyzed using the Kaplan-Meier method. The black solid and dotted lines indicate the stratified post-treatment WFA^+^-M2BP values with a COI ≤ 2.0 and a COI > 2.0, respectively. The incidence rate differed significantly between the two groups (*P* < 0.0001 by the log-rank test). The numbers of patients at risk at each time point are shown below the graphs.

Fig 2 shows the relation between the cumulative incidence of HCC and the post-Tx WFA^+^-M2BP values, stratified by age. In the patients with age > 60 years, the 5- and 10-year cumulative risks of HCC were 27.3% and 45.5% for the post-Tx WFA^+^-M2BP > 2.0 COI group. On the other hand, in the patients with age ≤ 60 years, the 5- and 10-year cumulative risks of HCC were 1.3% and 2.6% for the post-Tx WFA^+^-M2BP ≤ 2.0 COI group. There were significant differences in HCC incidence between the post-Tx WFA^+^-M2BP > 2.0 COI group and post-Tx WFA^+^-M2BP ≤ 2.0 COI group for both age categories (*P* < 0.0001 by the log-rank test).

**Fig 3:**
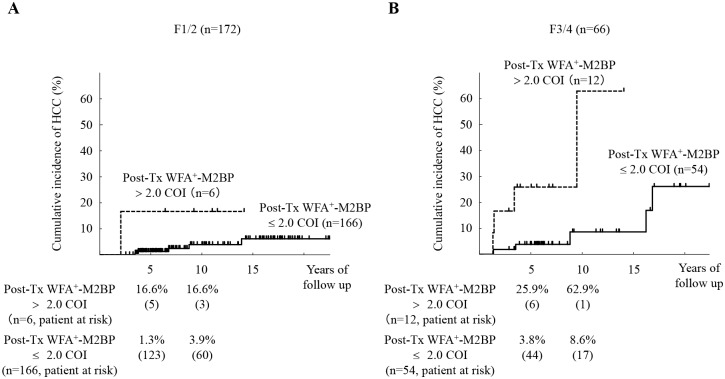
Cumulative incidence of hepatocellular carcinoma (HCC) according to post-treatment WFA^+^-M2BP values, stratified by stage of fibrosis. (A): F1/2 (n = 172). (B): F3/4 (n = 66). Cumulative incidences of HCC according to post-treatment WFA^+^-M2BP values were analyzed using the Kaplan-Meier method. The black solid and dotted lines indicate the stratified post-treatment WFA^+^-M2BP values with a COI ≤ 2.0 and a COI > 2.0, respectively. There were no significant differences in HCC incidence with F1/2 group (*P* = 0.09 by the log-rank test). On the other hand, the incidence rate differed significantly with F3/4 group (*P* < 0.01 by the log-rank test). The numbers of patients at risk at each time point are shown below the graphs.

Fig 3 shows the relation between the cumulative incidence of HCC and the post-Tx WFA^+^-M2BP values, stratified by stage of fibrosis. In the patients with F3/4, the 5- and 10-year cumulative risks of HCC were 25.9% and 62.9% for the post-Tx WFA^+^-M2BP > 2.0 COI group. On the other hand, in the patients with F1/2, the 5- and 10-year cumulative risks of HCC were 1.3% and 3.9% for the post-Tx WFA^+^-M2BP ≤ 2.0 COI group. There were significant differences in HCC incidence between the post-Tx WFA^+^-M2BP > 2.0 COI group and post-Tx WFA^+^-M2BP ≤ 2.0 COI group with advanced fibrosis (F3/4) patients (*P* < 0.01 by the log-rank test).

### Predictive value of HCC incidence versus WFA^+^-M2BP and AFP

Table 3 shows the AUROC analyses for prediction of the development of HCC at 3, 5 and 10 years with AFP and WFA^+^-M2BP. The post-Tx WFA^+^-M2BP was superior to the post-Tx AFP for predicting the development of HCC at each of 3, 5 and 10 years.

**Table 3:** Areas under the Curve for Censored Development of HCC at 3, 5, and 10 Years in the WFA^+^-M2BP and AFP Group.

Factors	3 years	5 years	10 years
Pre-Tx AFP (ng/mL)	0.678 (0.508–0.849)	0.710 (0.596–0.825)	0.620 (0.482–0.758)
Post-Tx AFP (ng/mL)	0.884 (0.833–0.934)	0.782 (0.644–0.919)	0.631 (0.445–0.816)
Pre-Tx WFA^+^-M2BP (COI)	0.603 (0.331–0.874)	0.621 (0.410–0.831)	0.604 (0.443–0.764)
Post-Tx WFA^+^-M2BP (COI)	0.909 (0.788–1.000)	0.812 (0.670–0.955)	0.707 (0.545–0.868)

Abbreviations: AFP, α-fetoprotein; WFA^+^-M2BP, *Wisteria floribunda* agglutinin-positive human Mac-2 binding protein.

### Chronological changes in the WFA^+^-M2BP and AFP values after IFN treatment

In the 238 patients with SVR, the median values of the chronological change in WFA^+^-M2BP at pre-Tx and post-Tx were 1.70 (range: 0.28 to 12.04 COI) and 0.80 (range: 0.17 to 5.29 COI). The post-Tx WFA^+^-M2BP values were significantly decreased relative to the pre-Tx WFA^+^-M2BP values (*P* < 0.001).

**Fig 4:**
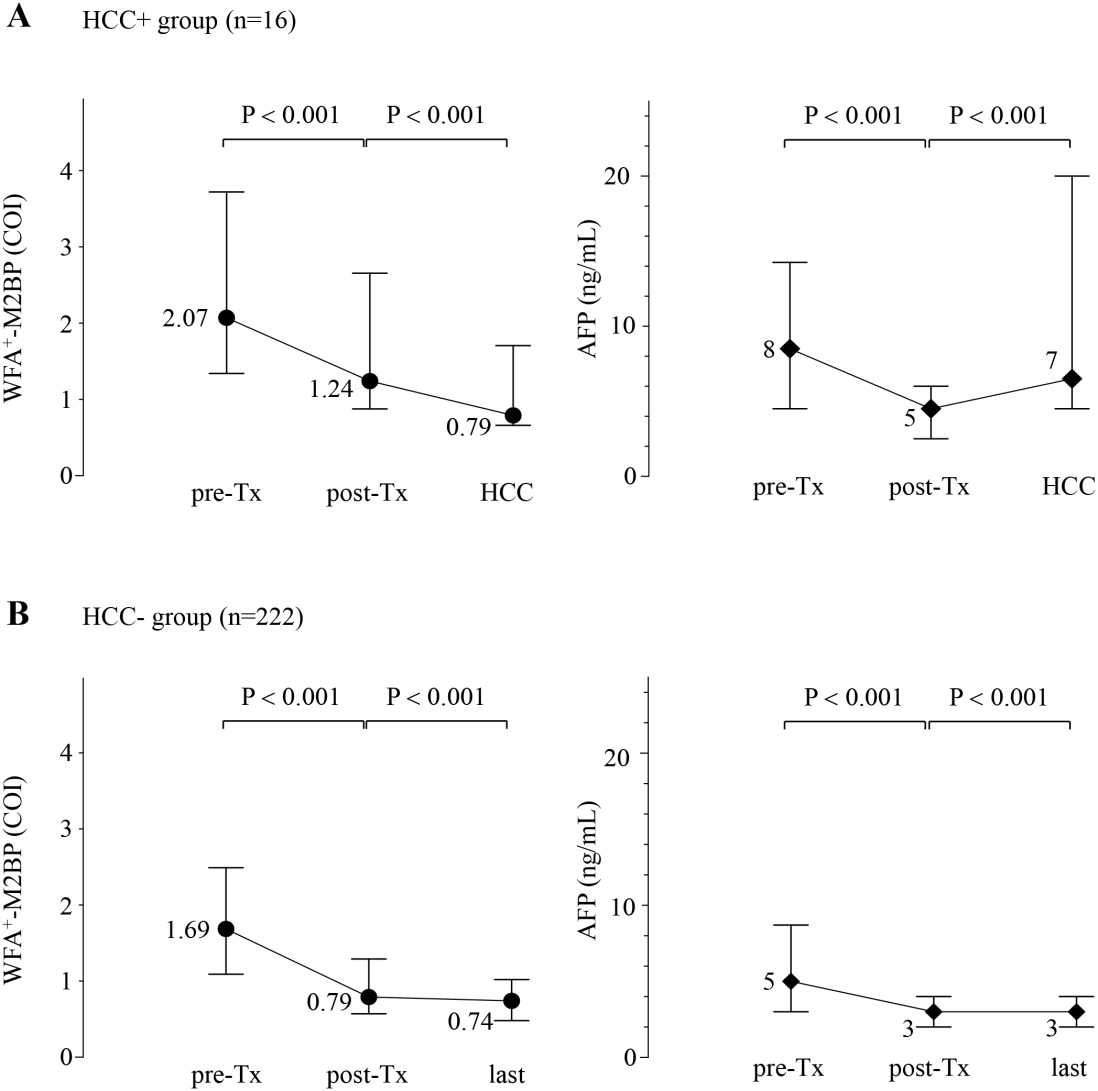
Chronological changes in the WFA^+^-M2BP and AFP values at pre-treatment, post-treatment, the time of HCC development, and the last visit of the 238 patients with sustained virological response. Dots represent the median serum WFA^+^-M2BP values at each time point, and the error bar represents the interquartile range. Diamonds represent the median serum AFP values at each time point, and the error bar represents the interquartile range. (A): Patients who developed HCC (n = 16). WFA^+^-M2BP values were decreased at post-treatment and increased at HCC development. And AFP values were decreased at post-treatment and increased at HCC development. (B): Patients who did not developed HCC (n = 222). WFA^+^-M2BP values were decreased at post-treatment and last clinical visit. And AFP values were decreased at post-treatment and last clinical visit.

Next, we analyzed the WFA^+^-M2BP and AFP values in the 16 patients who developed HCC. Fig 4A shows the chronological changes in WFA^+^-M2BP and AFP values at pre-Tx, post-Tx, and the time of HCC development for the 16 patients. The median WFA^+^-M2BP values of the 16 patients who developed HCC at pre-Tx, post-Tx and the time of HCC development were 2.07 (range: 0.99 to 8.04 COI), 1.24 (range: 0.42 to 4.44 COI) and 0.79 (range: 0.41 to 2.79 COI). The median AFP values of 16 patients at pre-Tx, post-Tx and the time of HCC development were 8 (range: 2 to 63 ng/mL), 5 (range: 1 to 7 ng/mL) and 7 (range: 3 to 5463 ng/mL). The WFA^+^-M2BP values at the time of post-Tx were significantly lower than those at the time of pre-Tx (*P* < 0.001). Additionally, the WFA^+^-M2BP values at the time of HCC development were significantly lower than those at the time of post-Tx (*P* < 0.001). The AFP values at the time of post-Tx were significantly lower than those at the time of pre-Tx (*P* < 0.001). In contrast, the AFP values at the time of HCC development were significantly higher than those at the time of post-Tx (*P* < 0.001).

We also analyzed the WFA^+^-M2BP and AFP values in the 222 patients who did not develop HCC. The median WFA^+^-M2BP values of the 222 patients who did not develop HCC at pre-Tx, post-Tx and the last clinical visit were 1.69 (range: 0.28 to 12.04 COI), 0.79 (range: 0.17 to 5.29 COI) and 0.74 (range: 0.14 to 7.24 COI). The median AFP values of the 222 patients at pre-Tx, post-Tx and the last clinical visit were 5 (range: 1 to 200 ng/mL), 3 (range: 1 to 46 ng/mL) and 3 (range: 1 to 11 ng/mL). The WFA^+^-M2BP values at the time of post-Tx were significantly lower than those at the time of pre-Tx (*P* < 0.001). In addition, the WFA^+^-M2BP values at the time of last clinical visit were significantly lower than those at the time of post-Tx (*P* < 0.001). The AFP values at the time of post-Tx were significantly lower than those at the time of pre-Tx (*P* < 0.001). Similarly, the AFP values at the time of last clinical visit were significantly higher than those at the time of post-Tx (*P* < 0.001) (Fig 4B).

**Fig 5:**
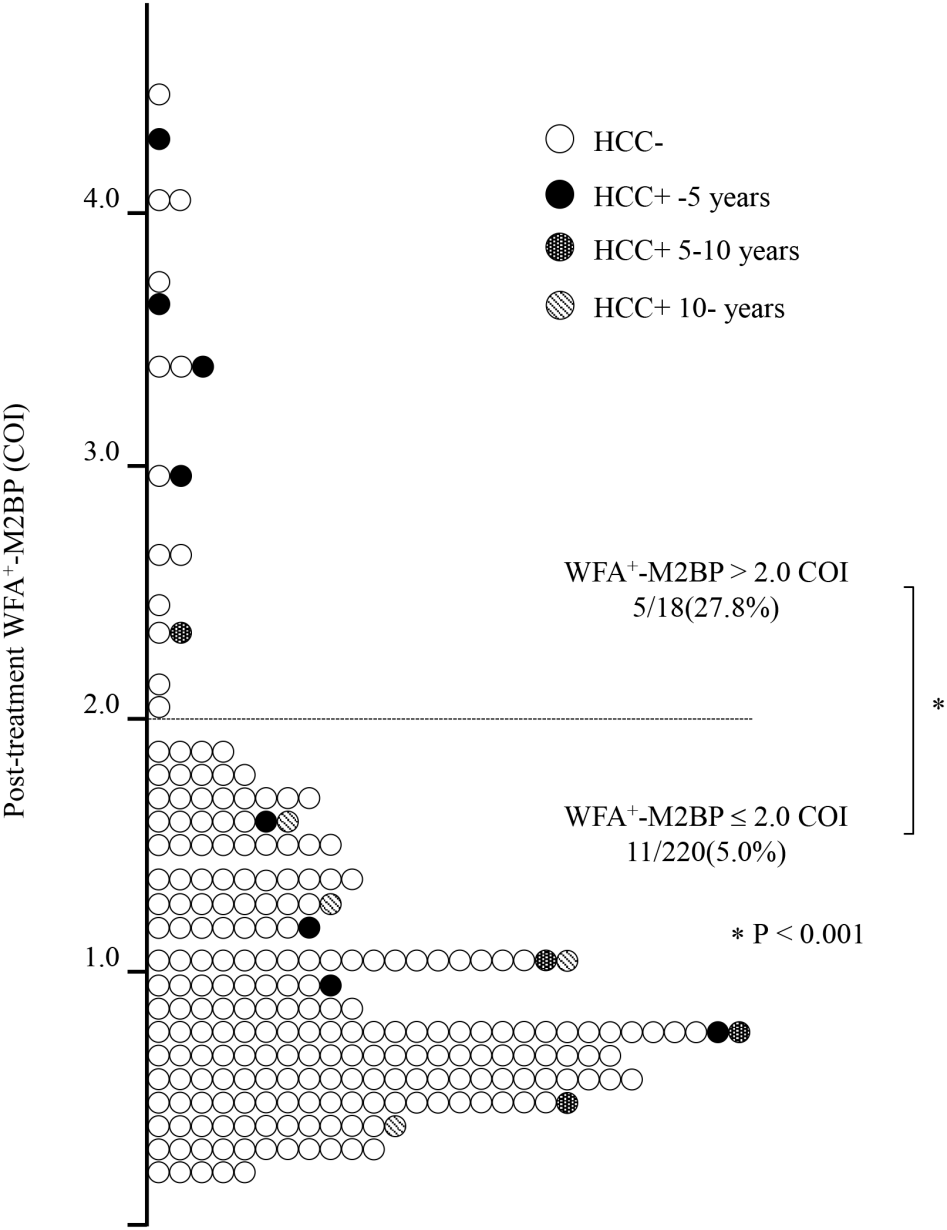
Relationship between post-treatment WFA^+^-M2BP values and HCC development. The distribution of WFA^+^-M2BP values was plotted. The dashed line indicates the 2.0 COI for WFA^+^-M2BP. The 16 patients who developed HCC were stratified according to the duration from SVR to HCC development in 5-year increments. Each time point is designated by a distinct symbol as indicated. 222 patients did not develop HCC, and 209 of these 222 patients (94.1%) were in the post-treatment WFA^+^-M2BP ≤ 2.0 COI group (white circles). During the follow-up period, 5 of 18 patients (27.8%) developed HCC in the post-treatment WFA^+^-M2BP > 2.0 COI group, which was significantly higher than the rate in the post-treatment WFA^+^-M2BP ≤ 2.0 COI group (5.0%, *P* < 0.001). In the post-treatment WFA^+^-M2BP > 2.0 COI group, 4 of 5 cases developed HCC within 5 years after IFN treatment (black circles).

Fig 5 shows the distribution of post-Tx WFA^+^-M2BP values. Among the 238 patients who achieved SVR, only 18 (7.6%) patients had post-Tx WFA^+^-M2BP > 2.0 COI. During the follow-up period, 5 patients (27.8%) developed HCC in the post-Tx WFA^+^-M2BP > 2.0 COI group (n = 18), and 4 of these 5 cases developed HCC within 5 years after IFN treatment. In contrast, 11 patients (5.0%) developed HCC in the post-Tx WFA^+^-M2BP ≤ 2.0 COI group (n = 220) (*P* < 0.001).

## Discussion

Previous studies have shown various risk factors for HCC development among patients with SVR: older age [14,15,16,18,19,33], male gender [19], heavy alcohol consumption [15,16], steatosis [33], advanced fibrosis [15,16,19], and lower platelet count [18]. In addition, recent studies reported that AFP values were significantly associated with HCC [18,27,34], and were also valuable for predicting future HCC risk after IFN treatment [35]. Asahina reported that post-IFN treatment AFP values were significantly associated with hepatocarcinogenesis [36]. In the present study, we specifically analyzed whether high WFA^+^-M2BP values might also be risk factors for HCC in patients with SVR.

In our study, the cumulative 5- and 10-year incidences of HCC were 3.4% and 7.5%. These results were consistent with previous studies, which reported that the cumulative incidences of HCC after SVR were 1.1–5.8% and 5.5–11.1% at 5 and 10 years [14,15,18,36–39], and the annual incidences were 0.37–1.21%/year [13,40,41].

The first main finding of our study was that the post-Tx WFA^+^-M2BP was selected as a new predictive marker for development of HCC among patients with SVR (Table 2). The values of WFA^+^-M2BP for predicting the development of HCC were determined to have a COI of 2.0 by time-dependent receiver operator characteristics (ROC) analysis. The cumulative incidence was significantly higher in the post-Tx WFA^+^-M2BP > 2.0 COI group. We were able to stratify the patients into different risk groups using the post-Tx WFA^+^-M2BP values and another simple risk factor, for example, age (Fig 2). Older age has been reported to confer a risk for hepatocellular carcinoma [14], which is an important association in Japan due to the aging of the population. Moreover, the post-Tx WFA^+^-M2BP values were significant predictor for HCC among patients with F3/4. Cumulative incidence of HCC was significantly higher in patients with higher post-Tx WFA^+^-M2BP values when patients were stratified by the stage of fibrosis (Fig 3). The post-Tx WFA^+^-M2BP values are not just a marker for liver fibrosis. Elevation of post-Tx WFA^+^-M2BP values as a potential risk for hepatocarcinogenesis with advanced fibrosis. And the time-dependent AUROC analysis suggested that WFA^+^-M2BP is superior to AFP as a predictor for the development of HCC.

The second main finding of our study was that the WFA^+^-M2BP values were decreased in patients who achieved SVR, even in those who developed HCC. Kuno previously reported that WFA^+^-M2BP values were decreased by IFN treatment [20]; to this result we added that the WFA^+^-M2BP values were decreased even in our IFN-treated patients who achieved SVR. However, the post-Tx WFA^+^-M2BP values were significantly higher in the patients who developed HCC than in those who did not. This finding is particularly important because the post-Tx values of WFA^+^-M2BP have not been adequately evaluated. Our data are thus the first to demonstrate the distribution of WFA^+^-M2BP values at post-Tx.

The third main finding of our study was that AFP and WFA^+^-M2BP values manifested different behaviors between the time of post-Tx and HCC diagnosis in patients who developed HCC. Our previous paper reported a close association between AFP values and the stage of fibrosis [27], whereas another report showed an elevation in AFP values caused by necroinflammation injury and regeneration of the liver [42]. However, WFA^+^-M2BP values do not always correlate with the grade of hepatic activity as defined by HAI scoring of inflammation [20,28]. A slight elevation of post-Tx AFP values (> 5ng/mL) could indicate substantial risks for the development of HCC [36]. In the 16 patients who developed HCC in our study, AFP values were elevated from post-Tx to the time of HCC development. However, the WFA^+^-M2BP values decreased after SVR and decreased further at the time of HCC diagnosis (Fig 4). The Mac-2 binding protein is secreted from many cell types, including hepatocytes, and it has been shown to modulate many processes, particularly those related to cell adhesion [22,43,44]. Alterations in the quality and quantity of the Mac-2 binding protein have been observed during the progression of fibrosis [22–24]. Hepatic stellate cells are considered the main fibrogenic cell type of the liver [45,46]. Activation of hepatic stellate cells and reversal of hepatic stellate cell activation [47] might be associated with WFA^+^-M2BP values. WFA^+^-M2BP has been associated with changes in both the quality and quantity of the Mac-2 binding protein due to changes in glycosylation [20]. From these considerations, we think that the WFA^+^-M2BP values do not reflect the results of HCC development, but rather a pre-cancer status or hepatocellular carcinogenesis.

One of the limitations of the present study was its retrospective nature. A future prospective analysis will be needed to validate the efficacy of WFA^+^-M2BP as a predictor of HCC development. Another limitation is that we analyzed a relatively small number of HCC cases after SVR. Multi-center prospective registration of patients with SVR could overcome this deficiency.

Regardless of these limitations, this is the first report to describe the relationship between WFA^+^-M2BP values and HCC development after SVR. The rapid progress in the development of anti-viral agents [48,49] for hepatitis C suggests that the number of patients who achieve SVR—including elderly patients or patients with advanced fibrosis, who are regarded as being at high risk for HCC—might increase in the near future, especially in Japan. Therefore, the prediction of HCC development in patients with SVR is of increasing clinical relevance.

In conclusion, this study revealed an association between WFA^+^-M2BP and the risk of HCC development in patients with SVR. The results suggested that the WFA^+^-M2BP should not be limited to use in fibrosis stage screening but rather could be applied as a new predictor of HCC development after SVR.

## References

[1] Forner A, Llovet JM, Bruix J (2012) Hepatocellular carcinoma. Lancet 379: 1245–1255. 10.1016/S0140-6736(11)61347-0 22353262 PMC13537732

[2] Armstrong GL, Wasley A, Simard EP, McQuillan GM, Kuhnert WL, Alter MJ. (2006) The prevalence of hepatitis C virus infection in the United States, 1999 through 2002. Ann Intern Med 144: 705–714. 16702586 10.7326/0003-4819-144-10-200605160-00004

[3] Kiyosawa K, Umemura T, Ichijo T, Matsumoto A, Yoshizawa K, Gad A, et al (2004) Hepatocellular carcinoma: recent trends in Japan. Gastroenterology 127(Suppl): S17–26. 15508082 10.1053/j.gastro.2004.09.012

[4] Mohd Hanafiah K, Groeger J, Flaxman AD, Wiersma ST (2013) Global epidemiology of hepatitis C virus infection: new estimates of age-specific antibody to HCV seroprevalence. Hepatology 57: 1333–1342. 10.1002/hep.26141 23172780

[5] Thomas DL, Seeff LB (2005) Natural history of hepatitis C. Clin Liver Dis 9: 383–398. 16023972 10.1016/j.cld.2005.05.003

[6] Sherman M (2005) Hepatocellular carcinoma: epidemiology, risk factors, and screening. Semin Liver Dis 25: 143–154. 15918143 10.1055/s-2005-871194

[7] El-Serag HB, Rudolph KL (2007) Hepatocellular carcinoma: epidemiology and molecular carcinogenesis. Gastroenterology 132: 2557–2576. 17570226 10.1053/j.gastro.2007.04.061

[8] Singal AG, Volk ML, Jensen D, Di Bisceglie AM, Schoenfeld PS (2010) A sustained viral response is associated with reduced liver-related morbidity and mortality in patients with hepatitis C virus. Clin Gastroenterol Hepatol 8: 280–288,288.e1 10.1016/j.cgh.2009.11.018 19948249

[9] Pradat P, Tillmann HL, Sauleda S, Braconier JH, Saracco G, Thursz M, et al: HENCORE Group (2007) Long-term follow-up of the hepatitis C HENCORE cohort: response to therapy and occurrence of liver-related complications. J Viral Hepat 14: 556–563. 17650289 10.1111/j.1365-2893.2006.00829.x

[10] Bruno S, Stroffolini T, Colombo M, Bollani S, Benvegnù L, Mazzella G, et al: Italian Association of the Study of the Liver Disease (AISF) (2007) Sustained virological response to interferon-alpha is associated with improved outcome in HCV-related cirrhosis: a retrospective study. Hepatology 45: 579–587. 17326216 10.1002/hep.21492

[11] Backus LI, Boothroyd DB, Phillips BR, Belperio P, Halloran J, Mole LA. (2011) A sustained virologic response reduces risk of all-cause mortality in patients with hepatitis C. Clin Gastroenterol Hepatol 9: 509–516. 10.1016/j.cgh.2011.03.004 21397729

[12] Morgan RL, Baack B, Smith BD, Yartel A, Pitasi M, Falck-Ytter. (2013) Eradication of hepatitis C virus infection and the development of hepatocellular carcinoma: a meta-analysis of observational studies. Ann Intern Med 158: 329–337. 10.7326/0003-4819-158-5-201303050-00005 23460056

[13] Yoshida H, Shiratori Y, Moriyama M, Arakawa Y, Ide T, Sata M, et al (1999) Interferon therapy reduces the risk for hepatocellular carcinoma: national surveillance program of cirrhotic and noncirrhotic patients with chronic hepatitis C in Japan. IHIT Study Group. Inhibition of Hepatocarcinogenesis by Interferon Therapy. Ann Intern Med 131: 174–181. 10428733 10.7326/0003-4819-131-3-199908030-00003

[14] Asahina Y, Tsuchiya K, Tamaki N, Hirayama I, Tanaka T, Sato M, et al (2010) Effect of aging on risk for hepatocellular carcinoma in chronic hepatitis C virus infection. Hepatology 52: 518–527. 10.1002/hep.23691 20683951

[15] Tokita H, Fukui H, Tanaka A, Kamitsukasa H, Yagura M, Harada H, et al (2005) Risk factors for the development of hepatocellular carcinoma among patients with chronic hepatitis C who achieved a sustained virological response to interferon therapy. J Gastroenterol Hepatol 20: 752–758. 15853990 10.1111/j.1440-1746.2005.03800.x

[16] Iwasaki Y, Takaguchi K, Ikeda H, Makino Y, Araki Y, Ando M, et al (2004) Risk factors for hepatocellular carcinoma in Hepatitis C patients with sustained virologic response to interferon therapy. Liver Int 24: 603–610. 15566511 10.1111/j.1478-3231.2004.0956.x

[17] Kobayashi S, Takeda T, Enomoto M, Tamori A, Kawada N, Habu D, et al (2007) Development of hepatocellular carcinoma in patients with chronic hepatitis C who had a sustained virological response to interferon therapy: a multicenter, retrospective cohort study of 1124 patients. Liver Int 27: 186–191. 17311612 10.1111/j.1478-3231.2006.01406.x

[18] Ikeda M, Fujiyama S, Tanaka M, Sata M, Ide T, Yatsuhashi H, et al (2005) Risk factors for development of hepatocellular carcinoma in patients with chronic hepatitis C after sustained response to interferon. J Gastroenterol 40: 148–156. 15770398 10.1007/s00535-004-1519-2

[19] Makiyama A, Itoh Y, Kasahara A, Imai Y, Kawata S, Yoshioka K, et al (2004) Characteristics of patients with chronic hepatitis C who develop hepatocellular carcinoma after a sustained response to interferon therapy. Cancer 101: 1616–1622. 15378504 10.1002/cncr.20537

[20] Kuno A, Ikehara Y, Tanaka Y, Ito K, Matsuda A, Sekiya S, et al (2013) A serum "sweet-doughnut" protein facilitates fibrosis evaluation and therapy assessment in patients with viral hepatitis. Sci Rep 3: 1065 10.1038/srep01065 23323209 PMC3545220

[21] Narimatsu Y, Kuno A, Ito H, Kaji H, Kaneko S, Usui J, et al (2014) IgA nephropathy caused by unusual polymerization of IgA1 with aberrant N-glycosylation in a patient with monoclonal immunoglobulin deposition disease. PloS One 9: e91079 10.1371/journal.pone.0091079 24651839 PMC3961232

[22] Sasaki T, Brakebusch C, Engel J, Timpl R (1998) Mac-2 binding protein is a cell-adhesive protein of the extracellular matrix which self-assembles into ring-like structures and binds beta1 integrins, collagens and fibronectin. EMBO J 17: 1606–1613. 9501082 10.1093/emboj/17.6.1606 PMC1170508

[23] Iacovazzi PA, Trisolini A, Barletta D, Elba S, Manghisi OG, Correale M, et al (2001) Serum 90K/MAC-2BP glycoprotein in patients with liver cirrhosis and hepatocellular carcinoma: a comparison with alpha-fetoprotein. Clin Chem Lab Med 39: 961–965. 11758611 10.1515/CCLM.2001.155

[24] Artini M, Natoli C, Tinari N, Costanzo A, Marinelli R, Balsano C, et al (1996) Elevated serum levels of 90K/MAC-2 BP predict unresponsiveness to alpha-interferon therapy in chronic HCV hepatitis patients. J Hepatol 25: 212–217. 8878784 10.1016/s0168-8278(96)80076-6

[25] Cheung KJ, Tilleman K, Deforce D, Colle I, Van Vlierberghe H (2009) The HCV serum proteome: a search for fibrosis protein markers. J Viral Hepat 16: 418–429. 10.1111/j.1365-2893.2009.01083.x 19226329

[26] Kuno A, Sato T, Shimazaki H, Unno S, Saitou K, Kiyohara K, et al (2013) Reconstruction of a robust glycodiagnostic agent supported by multiple lectin-assisted glycan profiling. Proteomics Clin Appl 7: 642–647. 23640794 10.1002/prca.201300010

[27] Tateyama M, Yatsuhashi H, Taura N, Motoyoshi Y, Nagaoka S, Yanagi K, et al (2011) Alpha-fetoprotein above normal levels as a risk factor for the development of hepatocellular carcinoma in patients infected with hepatitis C virus. J Gastroenterol 46: 92–100. 10.1007/s00535-010-0293-6 20711614

[28] Yamasaki K, Tateyama M, Abiru S, Komori A, Nagaoka S, Saeki A, et al (2014) Elevated serum levels of WFA^+^-M2BP predict the development of hepatocellular carcinoma in hepatitis C patients. Hepatology 60: 1563–70. 10.1002/hep.27305 25042054 PMC4278450

[29] Desmet VJ, Gerber M, Hoofnagle JH, Manns M, Scheuer PJ (1994) Classification of chronic hepatitis: diagnosis, grading and staging. Hepatology 19: 1513–1520. 8188183

[30] Simmonds P, Holmes EC, Cha TA, Chan SW, McOmish F, Irvine B, et al (1993) Classification of hepatitis C virus into six major genotypes and a series of subtypes by phylogenetic analysis of the NS-5 region. J Gen Virol 74: 2391–2399. 8245854 10.1099/0022-1317-74-11-2391

[31] Ohno O, Mizokami M, Wu RR, Saleh MG, Ohba K, Orito E, et al (1997) New hepatitis C virus (HCV) genotyping system that allows for identification of HCV genotypes 1a, 1b, 2a, 2b, 3a, 3b, 4, 5a, and 6a. J Clin Microbiol 35: 201–207. 8968908 10.1128/jcm.35.1.201-207.1997 PMC229539

[32] Heagerty PJ, Lumley T, Pepe MS (2000) Time-dependent ROC curves for censored survival data and a diagnostic marker. Biometrics 56: 337–344. 10877287 10.1111/j.0006-341x.2000.00337.x

[33] Tanaka A, Uegaki S, Kurihara H, Aida K, Mikami M, Nagashima I, et al (2007) Hepatic steatosis as a possible risk factor for the development of hepatocellular carcinoma after eradication of hepatitis C virus with antiviral therapy in patients with chronic hepatitis C. World J Gastroenterol 13: 5180–5187. 17876888 10.3748/wjg.v13.i39.5180 PMC4171299

[34] Rodríguez-Díaz JL, Rosas-Camargo V, Vega-Vega O, Morales-Espinosa D, Mendez-Reguera A, Martínez-Tlahuel JL, et al (2007) Clinical and pathological factors associated with the development of hepatocellular carcinoma in patients with hepatitis virus-related cirrhosis: a long-term follow-up study. Clin Oncol 19: 197–203. 10.1016/j.clon.2006.12.005 17359907

[35] Di Bisceglie AM, Sterling RK, Chung RT, Everhart JE, Dienstag JL, Bonkovsky HL, et al (2005) Serum alpha-fetoprotein levels in patients with advanced hepatitis C: results from the HALT-C Trial. J Hepatol 43: 434–441. 16136646 10.1016/j.jhep.2005.03.019

[36] Asahina Y, Tsuchiya K, Nishimura T, Muraoka M, Suzuki Y, Tamaki N, et al (2013) α-fetoprotein levels after interferon therapy and risk of hepatocarcinogenesis in chronic hepatitis C. Hepatology 58: 1253–1262. 10.1002/hep.26442 23564522

[37] Hasegawa E, Kobayashi M, Kawamura Y, Yatsuji H, Sezaki H, Hosaka T, et al (2007) Efficacy and anticarcinogenic activity of interferon for hepatitis C virus-related compensated cirrhosis in patients with genotype 1b low viral load or genotype 2. Hepatol Res 37: 793–800. 17593231 10.1111/j.1872-034X.2007.00140.x

[38] Watanabe S, Enomoto N, Koike K, Izumi N, Takikawa H, Hashimoto E, et al: PERFECT Study Group (2011) Cancer preventive effect of pegylated interferon α-2b plus ribavirin in a real-life clinical setting in Japan: PERFECT interim analysis. Hepatol Res 41: 955–964. 10.1111/j.1872-034X.2011.00847.x 21707888

[39] Ogawa E, Furusyo N, Kajiwara E, Takahashi K, Nomura H, Maruyama, et al: Kyushu University Liver Disease Study (KULDS) Group (2013) Efficacy of pegylated interferon alpha-2b and ribavirin treatment on the risk of hepatocellular carcinoma in patients with chronic hepatitis C: a prospective, multicenter study. J Hepatol 58: 495–501. 10.1016/j.jhep.2012.10.017 23099187

[40] Shindo M, Hamada K, Oda Y, Okuno T (2001) Long-term follow-up study of sustained biochemical responders with interferon therapy. Hepatology 33: 1299–1302. 11343259 10.1053/jhep.2001.24100

[41] Kim KI, Sasase N, Taniguchi M, Mita K, Kim SR, Tanaka K, et al (2005) Prediction of efficacy of interferon treatment of chronic hepatitis C and occurrence of HCC after interferon treatment by a new classification. Intervirology. 48: 52–58. 15785090 10.1159/000082095

[42] Morgan TR, Ghany MG, Kim HY, Snow KK, Shiffman ML, De Santo JL, et al: HALT-C Trial Group (2010) Outcome of sustained virological responders with histologically advanced chronic hepatitis C. Hepatology 52: 833–844. 10.1002/hep.23744 20564351 PMC2932862

[43] Hu J, He J, Kuang Y, Wang Z, Sun Z, Zhu H, et al (2013) Expression and significance of 90K/Mac-2BP in prostate cancer. Exp Ther Med 5: 181–184. 23251263 10.3892/etm.2012.768 PMC3524163

[44] Sun L, Chen L, Sun L, Pan J, Yu L, Han L, et al (2013) Functional screen for secreted proteins by monoclonal antibody library and identification of Mac-2 Binding protein (Mac-2BP) as a potential therapeutic target and biomarker for lung cancer. Mol Cell Proteomics 12: 395–406. 10.1074/mcp.M112.020784 23184915 PMC3567862

[45] Bataller R, Brenner DA (2005) Liver fibrosis. J Clin Invest 115: 209–218. 15690074 10.1172/JCI24282 PMC546435

[46] Friedman SL (2008) Hepatic stellate cells: protean, multifunctional, and enigmatic cells of the liver. Physiol Rev 88: 125–172. 10.1152/physrev.00013.2007 18195085 PMC2888531

[47] Troeger JS, Mederacke I, Gwak GY, Dapito DH, Mu X, Hsu CC, et al (2012) Deactivation of hepatic stellate cells during liver fibrosis resolution in mice. Gastroenterology 143: 1073–1083. 10.1053/j.gastro.2012.06.036 22750464 PMC3848328

[48] Afdhal N, Zeuzem S, Kwo P, Chojkier M, Gitlin N, Puoti M, et al: ION-1 Investigators (2014) Ledipasvir and sofosbuvir for untreated HCV genotype 1 infection. N Engl J Med 15: 1889–1898. 10.1056/NEJMoa1402454 24725239

[49] Afdhal N, Reddy KR, Nelson DR, Lawitz E, Gordon SC, Schiff E, et al: ION-2 Investigators (2014) Ledipasvir and sofosbuvir for previously treated HCV genotype 1 infection. N Engl J Med 17: 1483–1493. 10.1056/NEJMoa1316366 24725238

